# Lyn Facilitates Glioblastoma Cell Survival under Conditions of Nutrient Deprivation by Promoting Autophagy

**DOI:** 10.1371/journal.pone.0070804

**Published:** 2013-08-02

**Authors:** Wei Michael Liu, Ping Huang, Niladri Kar, Monica Burgett, Gaelle Muller-Greven, Amy S. Nowacki, Clark W. Distelhorst, Justin D. Lathia, Jeremy N. Rich, John C. Kappes, Candece L. Gladson

**Affiliations:** 1 Department of Cancer Biology, The Lerner Research Institute, Cleveland Clinic, Cleveland, Ohio, United States of America; 2 Department of Quantitative Health Sciences, Cleveland Clinic, Cleveland, Ohio, United States of America; 3 Department of Stem Cell Biology and Regenerative Medicine, The Lerner Research Institute, Cleveland Clinic, Cleveland, Ohio, United States of America; 4 School of Biomedical Sciences, Kent State University, Kent, Ohio, United States of America; 5 Department of Medicine, Case Western Reserve University, Cleveland, Ohio, United States of America; 6 Department of Medicine, University of Alabama at Birmingham, Birmingham, Alabama, United States of America; The University of Chicago, United States of America

## Abstract

Members of the Src family kinases (SFK) can modulate diverse cellular processes, including division, death and survival, but their role in autophagy has been minimally explored. Here, we investigated the roles of Lyn, a SFK, in promoting the survival of human glioblastoma tumor (GBM) cells *in vitro* and *in vivo* using lentiviral vector-mediated expression of constitutively-active Lyn (CA-Lyn) or dominant-negative Lyn (DN-Lyn). Expression of either CA-Lyn or DN-Lyn had no effect on the survival of U87 GBM cells grown under nutrient-rich conditions. In contrast, under nutrient-deprived conditions (absence of supplementation with L-glutamine, which is essential for growth of GBM cells, and FBS) CA-Lyn expression enhanced survival and promoted autophagy as well as inhibiting cell death and promoting proliferation. Expression of DN-Lyn promoted cell death. In the nutrient-deprived GBM cells, CA-Lyn expression enhanced AMPK activity and reduced the levels of pS6 kinase whereas DN-Lyn enhanced the levels of pS6 kinase. Similar results were obtained *in vitro* using another cultured GBM cell line and primary glioma stem cells. On propagation of the transduced GBM cells in the brains of nude mice, the CA-Lyn xenografts formed larger tumors than control cells and autophagosomes were detectable in the tumor cells. The DN-Lyn xenografts formed smaller tumors and contained more apoptotic cells. Our findings suggest that on nutrient deprivation *in vitro* Lyn acts to enhance the survival of GBM cells by promoting autophagy and proliferation as well as inhibiting cell death, and Lyn promotes the same effects *in vivo* in xenograft tumors. As the levels of Lyn protein or its activity are elevated in several cancers these findings may be of broad relevance to cancer biology.

## Introduction

Lyn is one of eight members of the Src family of kinases (SFK) expressed in human cells [1]. The SFKs are highly homologous non-receptor cytoplasmic tyrosine kinases that modulate diverse cellular processes including adhesion, migration, division, death and survival [1]–[5]. Dysregulation of individual SFKs, including Lyn, occurs in several different types of tumor [4], [6]–[9]. Although the functions of SFKs appear to be influenced by the microenvironment as well as cell type and post-translational modifications [4], [6]–[9], little attention has been paid to the role of SFKs in promoting cell survival through regulation of autophagy. A potential role for SFKs in autophagy is suggested by the reports that Dasatinib, which inhibits multiple SFKs as well as Bcr-Abl, induces autophagy in multiple types of cancer cells, including GBM, under nutrient-rich conditions [10], [11]. In addition, c-Src has been shown to localize to autophagosomes in focal adhesion kinase (FAK)-deficient cells under nutrient-rich conditions [12].

Lyn activity is elevated in GBM, the highest grade of glioma tumors, as well as in breast cancer, acute myelocytic leukemia (AML), B-cell chronic lymphocytic leukemia (CLL) and Ewing’s sarcoma [13]–[19]. We found previously that Lyn activity and protein levels are elevated significantly in human biopsies of GBM [17], consistent with the earlier report that 15% of GFAP-v-Src transgenic mice spontaneously develop low-grade gliomas that progress to GBM tumors [20]. Neither gene amplification nor mutation of SFK genes appears to play a role in the elevated SFK activity in GBM or breast cancer cells (reviewed in [2], [21]).

Here, we investigated the role of Lyn in promoting survival of GBM cells under nutrient-rich conditions and conditions of nutrient deprivation focusing on its role in autophagy. During autophagy, cytoplasmic proteins and organelles are sequestered in autophagasomes, which allows the cell to generate energy and nutrients [22], [23], and autophagy is thought to play a role in tumor progression when the supply of nutrients is limited [22]. GBM cells, like many other cancers cells are addicted to glutamine and there is a growing body of evidence that glutamine plays a critical role in the metabolic reprogramming utilized by cancer cells to meet the demands of rapid proliferation and hypoxic conditions [24], [25]. Although glioblastoma tumors (GBM) are highly vascularized, the neovasculature is abnormal and tumor cell starvation and hypoxia can occur due to vascular thromboses and tumor necrosis [2]. To our knowledge no prior study has investigated the pro-survival function(s) of any SFKs in nutrient-deprived cells.

We found that the survival of GBM cells transduced with either a lentiviral vector carrying constitutively-active (CA) Lyn or a dominant-negative (DN) Lyn construct grown under nutrient-rich conditions did not differ from control cells. In contrast, the results of similar analyses carried out under conditions of nutrient deprivation indicated that Lyn promotes survival of nutrient-deprived GBM cells through both promotion of autophagy and inhibition of apoptosis. When grown as xenografts, the CA-Lyn tumor cells formed larger tumors that contained autophagosomes and DN-Lyn cells formed smaller tumors with increased evidence of apoptosis. These studies suggest that Lyn can play a role in promoting survival of GBM cells by facilitating autophagy and underscores the importance of gaining an improved understanding of SFK-associated mechanisms in stressed cells.

## Materials and Methods

### Ethics Statement

The animal experiments were performed in accordance with an approved protocol from the Cleveland Clinic Institutional Animal Care and Use Committee (IACUC) (#2011–0554).

### Cell Lines and Culture Conditions

U87 human GBM cells were obtained recently from the American Type Culture Collection and maintained in L-glutamine-free DMEM (Sigma Aldrich, D5030) supplemented with 10% FBS and 1 mM L-glutamine. SNB19 human GBM cells [26], a kind gift from Dr. Jasti Rao at the University of Illinois, Peoria, were maintained in Hams F12 medium (Sigma Aldrich, D6421) supplemented with 10% FBS and 1 mM L-glutamine [27]. The GBM cells were plated in this nutrient-rich medium and after 12 h, washed and then cultured in nutrient-deficient medium, *i.e*., L-glutamine-free DMEM supplemented with 1% BSA (Sigma Chemical Co.) and without FBS. GBM cells have an increased demand for L-glutamine [24], which suppresses autophagy in other cell types [28].

The primary human glioma stem cell (GSC) line (3832) [29] was cultured as neurospheres in nutrient-rich neural basal medium (NBM) containing EGF and bFGF [30].

In some experiments, inhibitors were added to the culture medium, *i.e.*, 4-amino-5-(4-chlorophenyl)-7-(dimethylethyl)pyrazolo[3,4-*d*]pyrimidine (PP2) (EMD Millipore), perifosine and rapamycin (Selleck Chemicals), 3-methylamine (3-MA), and chloroquine (Invitrogen).

### Generation of Lyn Construct-Expressing Lentiviral Vectors

CA-Lyn (Y508F) and DN-Lyn (Y397F) cDNAs were kind gifts from Dr. Evan Ingley at the Western Australian Institute for Medical Research [31]. They were subcloned into the multiple cloning site of the lentiviral vector (designated K072) that contains the puromycin selectable marker and green fluorescent protein (GFP) genes downstream of an internal ribosome entry site (IRES) [32]. The lentiviral vector, the packaging construct, and the vesicular stomatits virus G envelope (VSV-G) were transfected into 293T human embryo kidney cells to create infectious lentiviral vector-containing particles. Cells were transduced (≈5×10^6^ infectious particles/ml) with vectors expressing CA-Lyn, DN-Lyn, or empty vector. Stable populations of U87 and SNB19 cells were selected with 5 µg/ml puromycin (2 weeks), monitored for GFP fluorescence on a regular basis, and sorted for GFP-expressing cells as necessary. After transduction, GSCs were sorted by FACS based on GFP positivity.

### Antibodies

Antibodies were purchased as follows: rabbit anti-pY397FAK and anti-total FAK (Upstate Biotechnology, Inc.), rabbit anti-Fyn, anti-Lyn and anti-cSrc (Santa Cruz International); monoclonal antibody (mAb) anti-glyceraldehyde 3-phosphate dehydrogenase (GAPDH), and mAb anti-actin (Sigma Chemical Co.); rabbit anti-LC3B, anti-phospho-Akt and anti-total Akt, and antibodies to pS6 kinase and total S6 kinase (Cell Signaling); rabbit anti-LC3B (Nanotools); rabbit polyclonal IgG (sc-25575) (Santa Cruz Biotechnology); anti-phospho-AMPK (Thr172) and anti-total AMPK (Cell Signaling Technology) and anti-phospho-a1-AMPK (Thr172) (Millipore).

### Immunoprecipitation and Western Blot Analyses

Cells were lysed using lysis buffer containing NP-40 and protease inhibitors and electrophoresed or immunoprecipitated followed by western blotting and densitometry [27]. Activation of Lyn was assessed by immunoblotting to detect autophosphorylation of Y418 [1], [17].

### Viability, Cell Death, and Cell Cycle Analyses

Viability was assessed by trypan blue exclusion and apoptosis determined by FACS analysis of Annexin V-APC-stained cells, blotting for cleaved caspase-7, or TUNEL assays [6], [27], [33]. Cell cycle analysis was carried out by analysis of DNA content using a FACScan (BD Biosciences) [33]–[35]. Proliferation was assessed by EdU incorporation and FACS analysis [33].

### Detection and Quantification of Autophagosomes

Autophagosomes were detected by analysis of microtubule-associated protein light chain 3 (LC3) [23]. Lentiviral vector-transduced U87 cells expressing GFP were infected with red fluorescent protein (RFP)-LC3 lentivirus, fixed using buffered 4% paraformaldehyde and fluorescent puncta counted in at least 25 cells [23]. Primary human GSCs plated on laminin were fixed, permeabilized and reacted with anti-LC3 antibody followed by Alexa 594-conjugated secondary antibody [27]. Cells were viewed using a Leica DMRB 4000X microscope. In some experiments, the presence of autophagosomes or late autophagic compartments (autophagic vacuoles) were detected by transmission electron microscopy (EM), which was performed as described [36] using a FIT Tecnai G2 EM scope in the Core Facility at the Cleveland Clinic. Vesicles were counted as autophagosomes and late autophagic vacuoles only if they were limited by a double membrane and contained undegraded cytoplasm [36]. A random sampling of tumor cells was performed and examined by EM from 5 tumors expressing LV, 5 expressing DN-Lyn, and 4 expressing CA-Lyn.

### Animals and Treatments

Xenografting was performed as described previously [33]. Cells were injected with stereotactic assistance into the basal ganglia. On day 35 the mice were euthanized and the brains fixed in buffered-formalin or electron microscopy fixative followed by embedment in paraffin. All animal care, housing and procedures were in accordance with the guidelines and regulations established by the Animal Welfare Act (PL99–158) and the Guide of the Care and Use of Laboratory Animals as part of the fully accredited (American Association for the Accreditation of Laboratory Animal Care International) Animal Resources Program of the Cleveland Clinic (ARC08826). Method of Euthanasia: At euthanasia, mice were anesthetized with ketamine (100 mg/kg mouse weight i.p.) and xylazine (15 mg/kg mouse weight i.p.), and once a surgical plane of anesthesia was reached based on absence of palpebral and toe reflexes, the mice were euthanized by cervical dislocation. Immediately after euthanasia, the chest was opened and 20 ml of saline was infused with a pump into the heart, followed by 30 ml of 10% formalin or by 30 ml of cold 4% paraformaldehyde (for EM analysis).

### Immunohistochemistry

This was performed as described [27]. Intensity of staining was compared to that of the IgG negative control.

### Statistical Analysis

Statistical analyses were performed by the biostatistician and author (ASN). The statistical test utilized is stated in each figure legend.

## Results

### Expression of CA-Lyn Promotes Survival of Nutrient-Deprived GBM Cells

**Figure 1:**
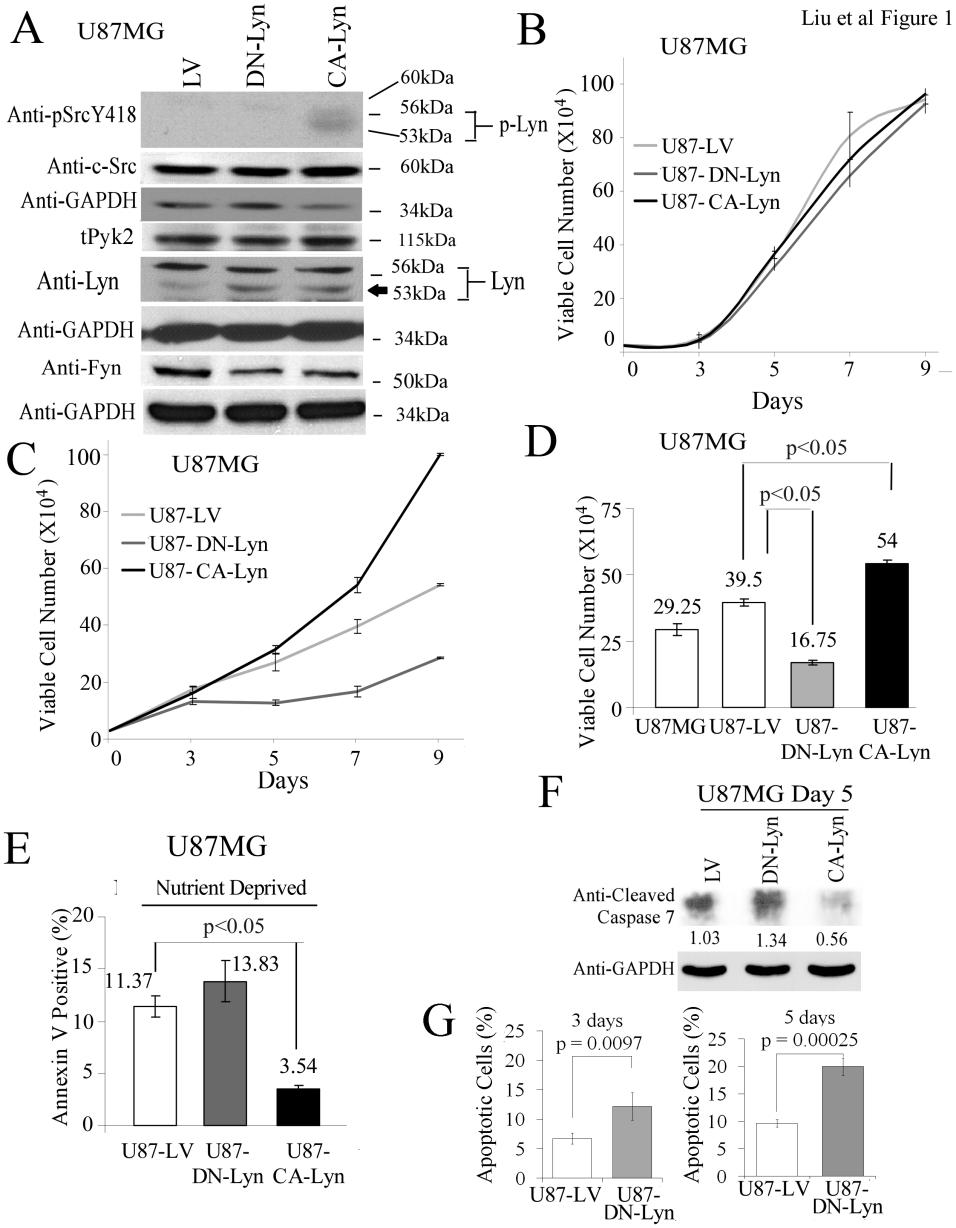
Expression of CA-Lyn promotes the survival of nutrient-deprived U87 human GBM cells. U87 human GBM cells expressing CA-Lyn, DN-Lyn or LV control were plated in DMEM with L-glutamine and 10% FBS. After 12 h, nutrient deprivation was induced by replacing the medium with L-glutamine- and FBS-free DMEM with 1% BSA. A, After 48 h of nutrient deprivation, whole cell lysates were western blotted with the indicated antibodies. B-D, After 7 days of nutrient deprivation, cell viability was determined by trypan blue exclusion (B–D) and apoptosis by FACs analysis of Annexin-V-labeled cells (E), blotting for cleaved caspase-7 (F), or FACS analysis for Annexin-V and propidium-iodide-labeled cells (G). B–E, and G, Conditions were assayed in replicas of 3 or 4, and the data analyzed and plotted as the mean±SEM. D & E, Statistical analysis using one-way ANOVA. G, Statistical analysis using t-test.

The levels of Lyn activity were manipulated by transducing U87 GBM cells with lentiviral vectors expressing a CA-Lyn construct or a DN-Lyn construct. The SFKs, including Lyn, are maintained in an inactive state by the C-terminal Src kinase (Csk), which phosphorylates the C-terminal negative regulatory peptide (Y508 in Lyn). The phosphorylated tyrosine binds to the SH2 domain folding SFK into an inactive configuration [1], [4]. Dephosphorylation of the negative regulatory peptide by tyrosine phosphatase-α or direct binding of the SH2 and SH3 domain to intracellular proteins such as FAK or activated tyrosine kinase growth factor receptors, allows the SFK structure to assume its active configuration [1], [4]. Constitutively active Lyn is generated by a Y508F mutation [1], [31]. We also utilized a DN-Lyn construct in which the autophosphorylation site is mutated (Y397F) [31] as we were unable to downregulate Lyn by >50% with a single shRNA lentiviral construct (WM Liu and CL Gladson, unpublished data). After expressing the constructs in U87 GBM cells using lentiviral vector, stable populations of cells were selected with puromycin, then sorted for GFP expression to maintain homogeneous, expression-positive populations. The expression of the constructs was confirmed by western blotting using anti-Lyn antibody, which demonstrated enhanced intensity of the band migrating at 53-kDa (5^th^ panel, Fig. 1A). Western blotting using an anti-Src(pY418) antibody further confirmed enhanced activity of Lyn in the U87-CA-Lyn cells as indicated by detection of a broad band migrating at 53–56-kDa and absence of Lyn activity in the U87-DN-Lyn cells (top panel, Fig. 1A). Only one other SFK, Lck, migrates on SDS PAGE at 53–56-kDa, but it is not expressed in human brain or GBM tumor tissue [17]. The SFK activity in control U87-LV cells was similar to that in parent U87 cells (data not shown) and no significant change in the expression of c-Src was detected in the U87-CA-Lyn or U87-DN-Lyn cells (Fig. 1A). Fyn protein was decreased by approximately 30% in U87 cells expressing CA-Lyn or DN-Lyn (Fig. 1A). No differences in the morphology of the control cells and those expressing the constructs were apparent.

**Figure 2:**
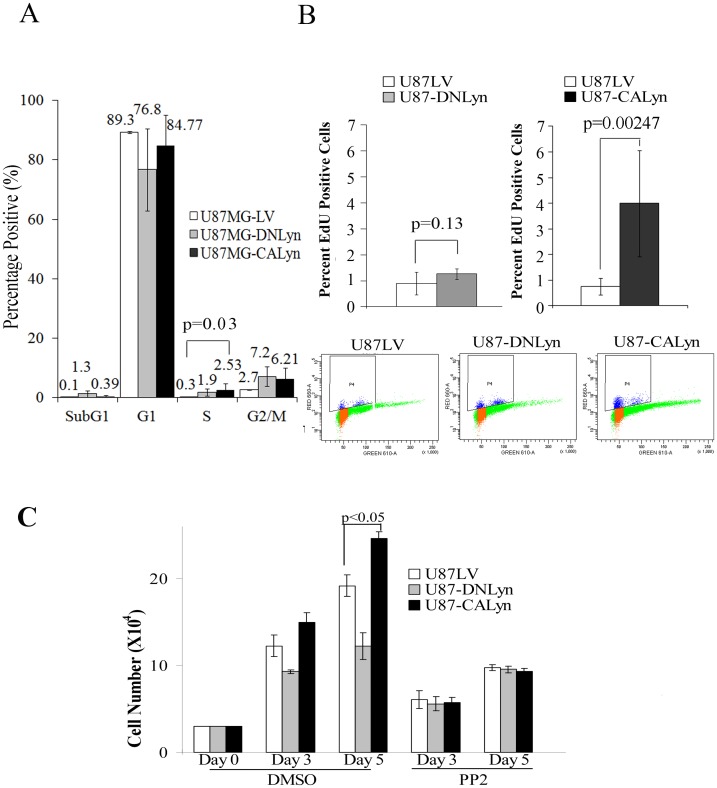
Expression of CA-Lyn increases EdU incorporation in nutrient-deprived U87 GBM cells. A–C, U87 cells expressing CA-Lyn, DN-Lyn or the LV control were plated and then starved of L-glutamine and FBS as described in Figure 1A. A, After 7 days of starvation cells were acetone fixed, stained with propidium iodide and analyzed for DNA content by FACS analysis. B, EdU was added after 41/2 days of nutrient deprivation and 18 hours later propidium-iodide was added and EdU incorporation analyzed by FACS. A & B, Conditions were assayed in replicas of 3 and the data analyzed as the mean±SD and plotted as a bar graph. C. 20 h after initiation of nutrient deprivation PP2 (200 nM) or DMSO (vehicle control) was added to the media. On day 5, viable adherent cells were counted by trypan blue exclusion. Conditions were performed in replicas of 3 or 4, and the data analyzed and presented as the mean±SEM.

Analysis of cell viability indicated that neither the expression of CA-Lyn nor DN-Lyn affected viability under nutrient-rich conditions (Fig. 1B). In contrast, under nutrient-deprivation conditions the viability of the U87-CA-Lyn cells was significantly greater and the viability of DN-Lyn cells significantly lower than that of U87-LV cells (Fig. 1C and D). Assessment of apoptosis by Annexin-V-staining (Fig. 1E), blotting for cleaved capsase-7 (Fig. 1F), or labeling for Annexin-V and propidium-iodide (Fig. 1G) showed that under nutrient-deprivation conditions the levels of apoptosis were significantly lower in the U87-CA-Lyn cells and significantly higher in the U87-DN-Lyn cells than the U87-LV cells (Fig. 1E-G). Annexin-V-labeling was not significantly different in the parent U87 and LV cells (data not shown). FACS analysis revealed a small increase in the percentage of U87 cells in S phase with expression of CA-Lyn as compared to the control LV cells (Fig. 2A). EdU incorporation studies also showed an increase in the percent of labeled CA-Lyn cells (4%) as compared to the control LV cells (1%) (Fig. 2B), which is consistent with CA-Lyn promoting proliferation. A small increase in the percentage of U87-DN-Lyn cells in sub-G1 (Fig. 2A) was detected on propidium-iodide labeling consistent with the increased apoptosis observed on Annexin-V-labeling as the percentage of apoptotic cells detected by cell cycle analysis (sub-G1) is typically smaller than that detected by Annexin-V-labeling [34], [35]. Taken together, these data suggested that Lyn plays an important role in regulating the survival of GBM cells propagated in the absence of L-glutamine and FBS.

To confirm that the kinase activity of CA-Lyn is required for Lyn promotion of GBM cell survival under nutrient deprivation, we utilized PP2 (a broad SFK inhibitor). We first selected the dosage of PP2 that inhibited SFK activity of the U87 cells when added to the media 24 h after initiation of nutrient deprivation. Blotting for pSrcY418 on day 5 confirmed inhibition of Lyn activity in the U87-CA-Lyn cells (Fig. S1A). In the presence of PP2, the survival of the nutrient-deprived U87-CA-Lyn cells was no different from that of U87-LV cells at days 3 and 5 (Fig. 2C), suggesting that SFK activity is necessary for the pro-survival effect of CA-Lyn.

### CA-Lyn Promotes Autophagy of Nutrient-Deprived GBM Cells

**Figure 3:**
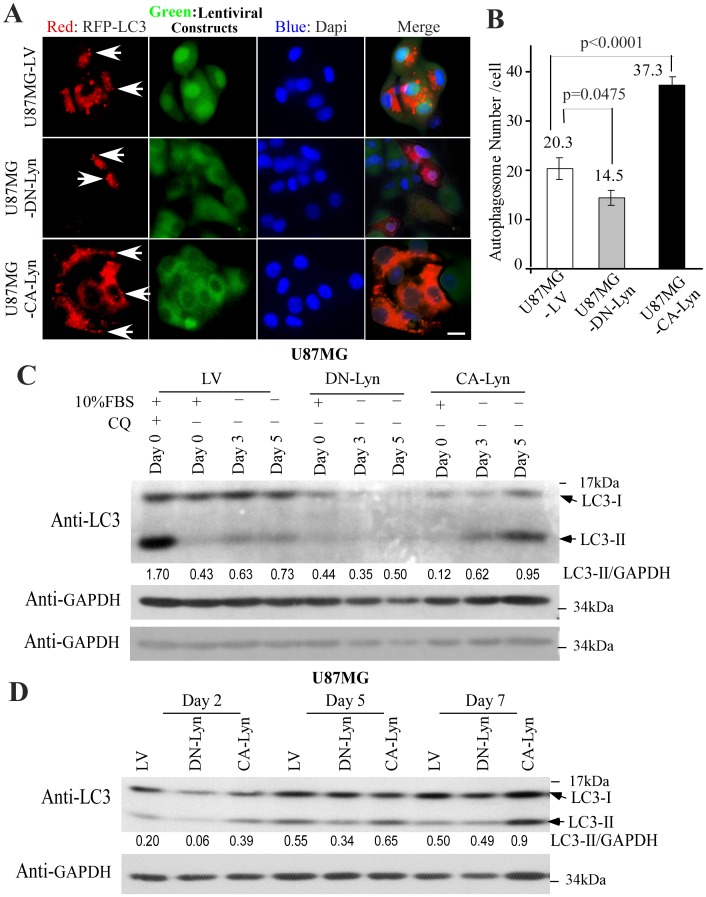
Expression of CA-Lyn increases the numbers of autophagosomes per cell and the levels of LC3B-II protein in nutrient-deprived U87 GBM cells. U87 GBM cells expressing CA-Lyn, DN-Lyn or the LV control and GFP downstream of the IRES were transduced with RFP-LC3 lentiviral vector then plated and subjected to nutrient deprivation as in Figure 1A. A-B, After 5 days, cells were fixed and stained with DAPI nuclear dye. Representative photomicrographs (Mag X40; Scale bar 10 µm) are shown in (A) with arrows indicating autophagosomes (puncta). The numbers of puncta per cell, counted in at least 25 cells for each construct, are shown in (B) in which data are presented as the mean ± SEM with analysis using one-way ANOVA. C-D, At the indicated time-points after initiation of nutrient deprivation, whole cell lysates were western blotted with the indicated antibodies.

We then transduced the cells with lentiviral vector expressing RFP-tagged LC3 [23] and cultured them under nutrient-deprivation conditions. On confocal microscopic examination, we found that the numbers of autophagosomes per cell were significantly higher in U87-CA-Lyn cells and significantly lower in U87-DN-Lyn cells than in U87-LV cells (Fig. 3A & B). Western blotting for LC3 protein further indicated the normalized levels of the lipid-modified LC3B-II protein, which is generated during autophagy, were higher in the U87-CA-Lyn cells than in the U87-LV cells at days 5 and 7 (Fig. 3C & D). At days 2, 3 and 5, lower levels of LC3B-II were present in the cells expressing DN-Lyn than in the U87-LV cells (Fig. 3C & D). The number of days required to detect autophagy under the experimental conditions used in these studies is likely due to the initial plating of the cells in nutrient-rich medium, but it also is possible that the GBM cells can synthesize L-glutamine as has been described for MCF7 cells [37].

Similar results were obtained using another human GBM cell line, SNB19. We found that lentiviral-mediated expression of CA-Lyn resulted in a broad band migrating at 53–56-kDa on blotting for active Src(pY418) (Fig. S1B) that was positive for Lyn on reprobing, and the levels of c-Src protein were not affected significantly (Fig. S1B). Fyn protein was decreased by approximately 30% in SNB19 cells expressing CA-Lyn or DN-Lyn (Fig. S1B). In addition, the level of normalized LC3B-II protein was higher in nutrient-deprived SNB19 cells expressing CA-Lyn at day 7 than in the SNB19 cells expressing the lentivirus vector alone (Fig. S1C).

**Figure 4:**
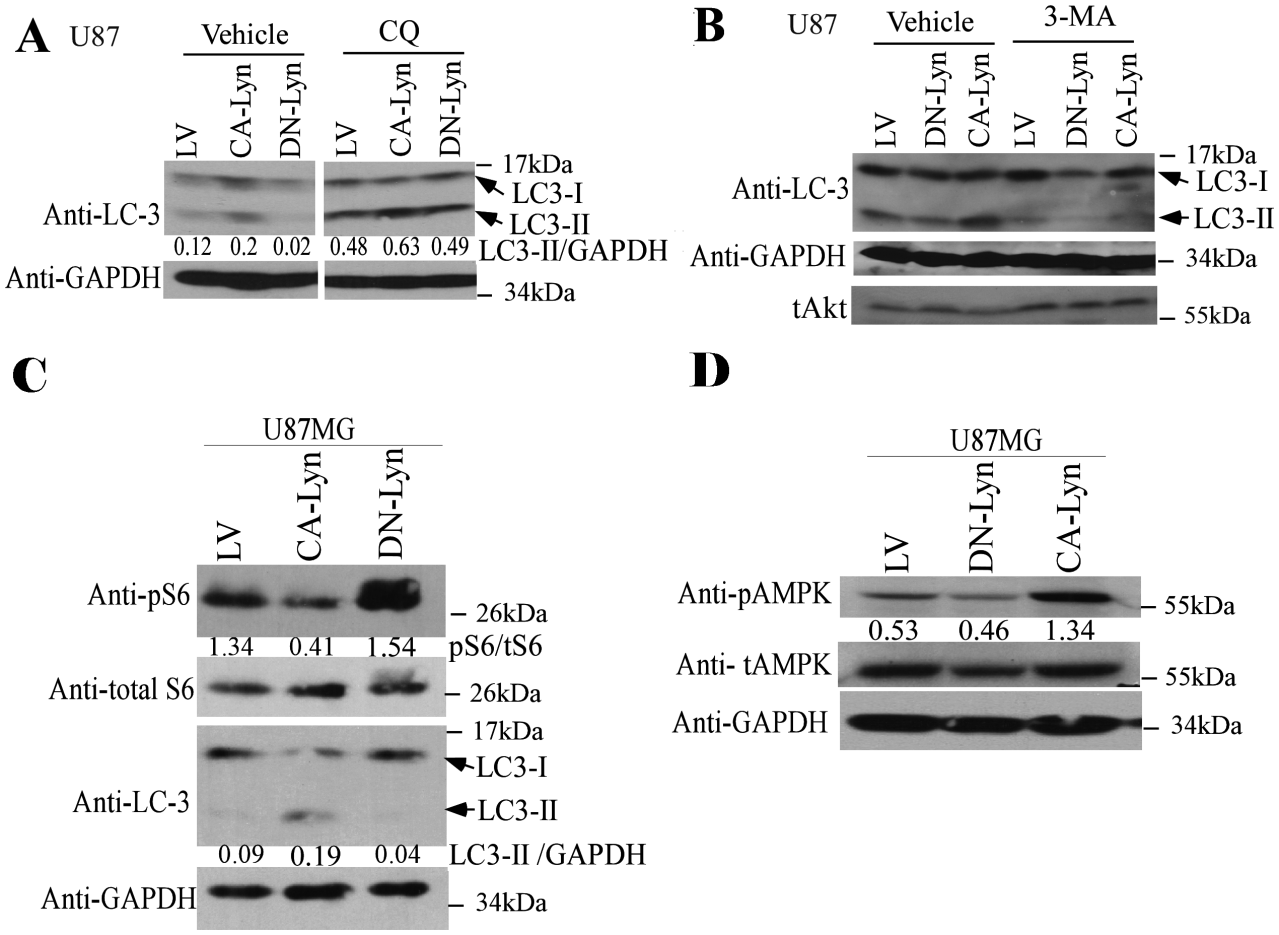
Expression of CA-Lyn increases levels of pAMPK in nutrient-deprived GBM cells. A-D, U87 GBM cells expressing CA-Lyn, DN-Lyn or the LV control were plated and subjected to nutrient deprivation as described in Figure 1A. A-B, 15 h prior to detergent lysis (day 5), cells were treated with vehicle, chloroquine (CQ; 10 µM) (A), or 3-methylamine (3-MA; 5 µM), and then whole cell lysates western blotted with the indicated antibodies. C & D, Cells were detergent lysed after five days of nutrient deprivation and blotted with the indicated antibodies. The densitometric readings for the LC3B-II band were normalized to GAPDH, the pS6 band normalized to total S6, and the pAMPK band normalized to total AMPK.

To confirm the effects of Lyn on autophagy in the nutrient-deprived cells we used several different approaches. Overnight treatment of nutrient-deprived cells with chloroquine, an inhibitor of lysosomal fusion, resulted in an increase in the levels of LC3B-II protein in the U87-CA-Lyn, U87-LV and U87-DN-Lyn cells (Fig. 4A), consistent with accumulation of lipid-modified LC3B-II protein on blockade of fusion of the autophagosome with the lysosome. Treatment with 3-MA, an upstream inhibitor of autophagy that blocks type II PI3K [22], caused a significant reduction in the levels of LC3B-II protein in U87-CA-Lyn, U87-LV and U87-DN-Lyn cells (Fig. 4B). Furthermore, treatment of the U87 cells expressing LV, CA-Lyn or DN-Lyn with chloroquine or 3-MA for 5 or 7 days under the same nutrient deprivation conditions resulted in a dramatic reduction in cell viability in all cell populations (Fig. S2), consistent with autophagy playing an important role in the CA-Lyn-associated increase in cell survival.

### Expression of CA-Lyn in Nutrient-Deprived GBM Cells is Associated with Enhanced AMPK Activity and a Reduction in the Levels of pS6 Kinase

As autophagy is negatively regulated by the mTORC1 complex, we estimated mTORC1 activity by assessing phosphorylation of S6 kinase, a downstream effector of mTORC1 [22], [23]. After 5 days of nutrient-deprivation, the levels of pS6 kinase were lower in U87-CA-Lyn and higher in U87-DN-Lyn cells than in U87-LV cells (Fig. 4C), consistent with the observed differences in autophagosome number and LC3B-II protein levels at this time point (Fig. 3A-D). Moreover, treatment with rapamycin, an inhibitor of mTORC1, resulted in higher levels of LC3B-II protein in nutrient-deprived U87-CA-Lyn cells than in vehicle-treated cells (Fig. S3A).

Akt activation of mTORC1 can inhibit autophagy [22]. The levels of pAkt(S308) in nutrient-deprived U87-CA-Lyn cells were unchanged at days 5 and 7 when the cells were undergoing autophagy (Fig. S3B); however, there was a transient increase in pAkt at day 2, prior to signs of autophagy (Fig. S3B). The levels of pAkt in the nutrient-deprived U87-DN-Lyn cells remained constant (Fig. S3B). Perifosine, an Akt inhibitor, enhanced the levels of LC3B-II protein in all cell populations (Fig. S3A). Others have reported that generalized SFK inhibition in osteosarcoma cells results in apoptosis, which was attributed to inhibition of the FAK/p130CAS signaling axis [38]; however, we did not find any changes in FAK activity (pY397 and pY925) with expression of CA-Lyn or DN-Lyn in the nutrient-deprived U87 or SNB19 GBM cells (Fig. S3C and data not shown). Nutrient deprivation is known to activate AMPK [22], and we found higher levels of pAMPK in nutrient-deprived U87-CA-Lyn cells than in nutrient-deprived U87-LV cells (Fig. 4D). There was no significant difference in the level of pAMPK in nutrient-deprived U87-DN-Lyn cells as compared to U87-LV cells.

### Expression of CA-Lyn in Nutrient-Deprived Primary Human Glioma Stem Cells Increases Autophagy

GSCs are thought to be the tumor-initiating cell in GBM [39]. We therefore transduced primary human GSCs with GFP-CA-Lyn, GFP-DN-Lyn or GFP-LV, sorted for GFP-expressing cells, and plated the cells on laminin in NBM. Higher Src activity was confirmed in the GSCs expressing CA-Lyn than those expressing LV (data not shown). Immunofluorescent staining for LC3 protein in the GSCs starved of EGF and bFGF for 6 h indicated that the numbers of autophagosomes per cell was significantly higher in the GSCs expressing CA-Lyn as compared to those expressing LV, and significantly lower in the GSCs expressing DN-Lyn as compared to those expressing LV (Fig. S4A & B). We also found higher pAMPK levels in the GSCs expressing CA-Lyn as compared to LV (Fig. S4C), suggesting that increased AMPK activity relieves the inhibition of mTORC1 on autophagy.

### Expression of CA-Lyn Promotes the Survival of GBM Cells Propagated as Intracerebral Xenografts and Promotes Autophagy in the Tumor Cells

**Figure 5:**
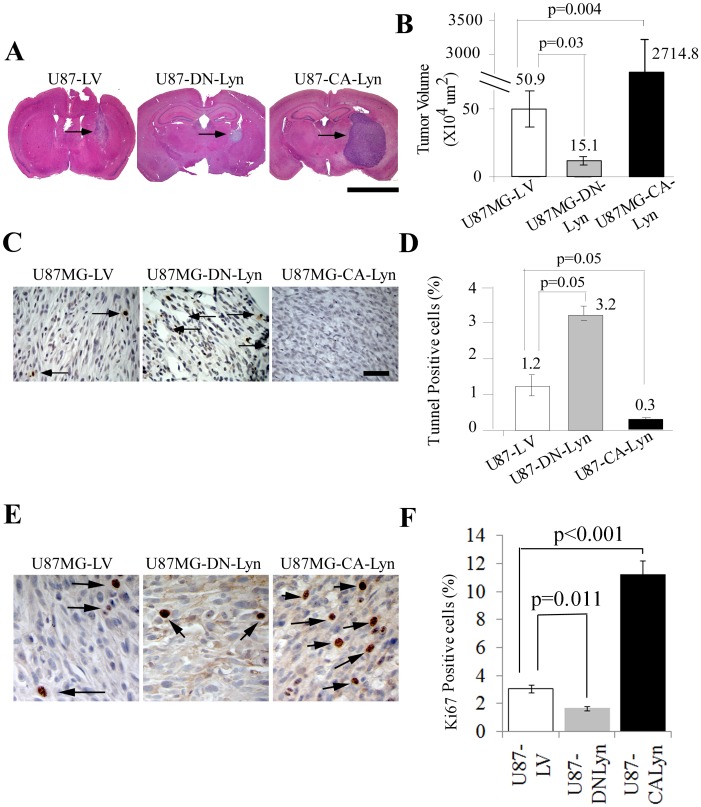
Expression of CA-Lyn in GBM cells results in significantly larger tumors whereas expression of DN-Lyn results in smaller tumors. Cells were harvested, resuspended in PBS, and 1×10^5^ cells in 5 µl injected with stereotactic assistance into the nude mouse brain. At 35 days, mice were euthanized, brains harvested, fixed and analyzed. (A, B) Representative H&E sections of tumors generated on injection of U87 cells expressing control LV, CA-Lyn or DN-Lyn (n = 6/group) are shown in (A). Arrows denote the tumors (Mag X1.25; Scale bar 5 mm). Tumor volumes for each group are presented as the mean±SEM and analyzed using one-way ANOVA in (B). C-D, Representative TUNEL-stained tumor sections are shown in (C) (Scale bar 50 µm). Arrows denote TUNEL-positive cells. TUNEL-positive cells were counted in at least 2 slides for each tumor and the percentage of TUNEL-positive cells is presented as the mean ± SEM with analysis using a Mann Whitney test (D). E-F, Ki67 staining of tumor sections is shown (Mag X40) in (E). Percentage of Ki67 positive cells is presented as the mean±SEM with analysis using one-way ANOVA (F).

To determine whether Lyn promotes the survival of GBM cells *in vivo*, we injected U87-CA-Lyn, U87-DN-Lyn and U87-LV cells into the brains of nude mice. After 35 days, the U87-CA-Lyn cells had formed significantly larger tumors than the U87-LV cells whereas the U87-DN-Lyn cells had formed significantly smaller tumors (Fig. 5A & B). GFP expression was confirmed in tumor cells in all tumors (Fig. S4D). As compared to the U87-LV tumors, there were significantly fewer TUNEL-positive cells in the U87-CA-Lyn tumors and a significantly higher number of TUNEL-positive cells in the U87-DN-Lyn tumors (Fig. 5C & D), which was consistent with our *in vitro* findings when cells were propagated as a monolayer under nutrient-deprivation conditions (Fig. 1E & F). Detection of proliferating cells by Ki67 mAb labeling showed a significant increase in the CA-Lyn versus the LV tumors, indicating that the larger tumor size with expression of CA-Lyn was due in part to increased proliferation (Fig. 5E & F). Ki67 labeling of the DN-Lyn tumors showed a significant decrease as compared to the LV tumors (Fig. 5E & F).

**Figure 6:**
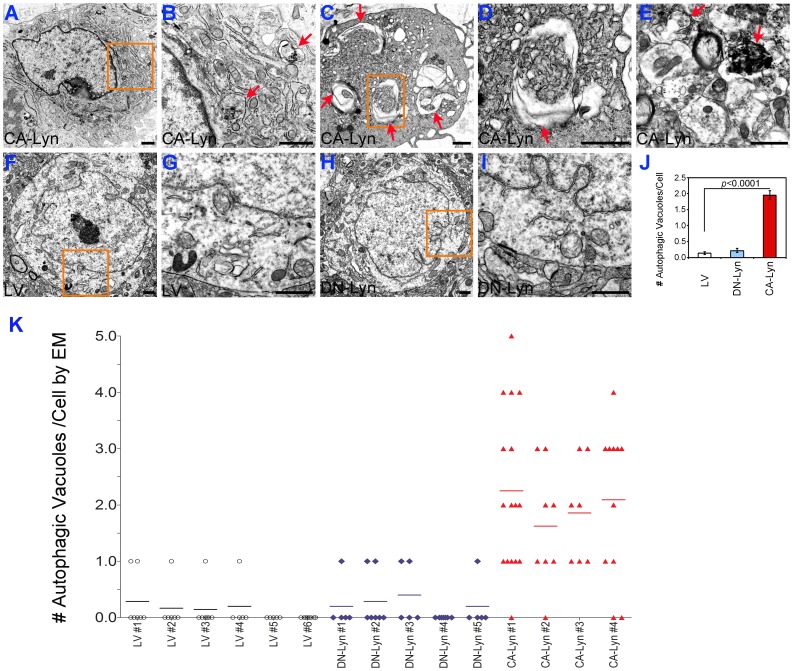
Expression of CA-Lyn in GBM cells results in larger tumors containing autophagic vacuoles. Xenograft tumors expressing LV, CA-Lyn or DN-Lyn were generated as described in the legend for Figure 5 and after euthanasia on day 35 tumors were fixed in EM fixative, followed by transmission EM as described in the Materials and Methods. A-E, Three different representative cells from CA-Lyn tumors; panel B-higher magnification of the box in Panel A, and panel D-higher magnification of the box in panel C. F & G, Representative tumor cell from LV tumor; panel G-higher magnification of the box in panel F. H & I, Representative tumor cell from DN-Lyn tumor; panel I-higher magnification of the box in panel H. Scale bars in each panel denote 1 µm. Arrows denote examples of autophagic vacuoles. J, Quantitation of autophagic vacuoles/tumor cell shows a significant increase in the CA-Lyn tumors as compared to the LV tumors (p value <0.0001; t-test). K, Scattergram denoting the number of autophagic vacuoles/tumor cell.

To evaluate autophagy in the xenograft tumors we utilized EM analysis. Representative photomicrographs of autophagic vacuoles (autophagosomes and late autophagic compartments) in tumor cells and the results of quantitation are shown in Figure 6A-K. These experiments revealed that while the occurrence of autophagic vacuoles in the U87-LV tumors (Fig. 6F & G) and U87-DN-CA tumors (Fig. 6H & I) was uncommon, autophagic vacuoles were present in most U87-CA-Lyn tumor cells examined (Fig. 6A-E, J & K). Collectively, these data indicate that Lyn promotes the survival of GBM cells in vivo through promotion of autophagy and proliferation, as well as inhibition of cell death.

## Discussion

Here, we show that Lyn promotes the survival of nutrient-stressed human GBM cell lines and primary human GSCs by promoting autophagy and proliferation as well as inhibiting cell death. The mechanism underlying the pro-autophagy effect of Lyn in the nutrient-deprived U87 GBM tumor cells and in the growth factor-deprived GSCs *in vitro* correlated with increased activation of AMPK and inhibition of the mTORC1 complex. The decrease in phosphorylation of S6 kinase in the GBM cells expressing CA-Lyn and the increase in the cells expressing DN-Lyn was consistent with the effects of these constructs on autophagy. Glucose, growth factor and amino acid deprivation activate AMPK thereby relieving mTORC1 inhibition of autophagy [22]. Consistent with this, nutrient-deprivation was associated with an increase in pAMPK in the GBM cells and GSCs expressing CA-Lyn. Although in most cell types the PI3K/Akt axis signals to activate mTORC1 inhibition of autophagy, the lack of change in Akt activity in the time frame in which autophagy was detectable (days 5 and 7) suggests that it may not be involved in Lyn regulation of mTORC1 activity. Currently, we cannot rule out the possibility that mTORC2 plays a role as there is evidence that it also can regulate autophagy in glioma cells [40].

SFK activity was necessary for the prosurvival effect of Lyn in nutrient-deprivation conditions, which suggests that Lyn kinase activity is necessary. However, we have not yet identified its substrate. Although multiple SFK substrates have been identified, none are known to positively regulate autophagy. The tyrosine phosphorylation of the known SFK substrates, FAK(Y397 and Y925) (Fig. S3C), cortactin or N-WASP (WM Liu and CL Gladson, unpublished observations) in the U87-CA-Lyn or U87-DN-Lyn cells was not affected after 5 days of nutrient deprivation.

The manipulation of Lyn activity also affected apoptosis and proliferation of nutrient-deprived cells. Expression of CA-Lyn resulted in a significant reduction in cell death and significant increase in EdU incorporation whereas expression of DN-Lyn resulted in a significant increase in cell death but no change in EdU incorporation. Although miRNA-205 has been shown to inhibit mRNA and protein expression of Lyn, c-Src, and c-Yes in A498 cells resulting in G0/G1 cell cycle arrest and apoptosis [41], and Lyn specifically reduced expression of miRNA-181b that represses the anti-apoptotic protein Mcl1 [42], we did not detect a change in miRNA expression (≥ or ≤1.5-fold) in the U87-CA-Lyn or U87-DN-Lyn cells as compared to U87-LV cells using the Human Cancer miRNA PCR Array (MAH-102A, Qiagen) (WM Liu and CL Gladson, unpublished data). Similarly, although SFKs can promote survival through the FAK/p130CAS or PI3K/Akt pro-survival pathways [38], [43], neither FAK nor Akt activity was altered in the nutrient-deprived GBM cells expressing CA-Lyn or DN-Lyn. It is possible that the cellular localization of CA-Lyn [44], [45], the cell surface binding partner of Lyn [46], or Lyn phosphorylation of the growth arrest and DNA damage protein 34 (GADD34) [47] contribute to the anti-death effect in GBM but this remains to be elucidated.

Our *in vivo* studies in which GBM cells expressing DN-Lyn were propagated in the nude mouse brain showed a significant reduction in tumor size that was associated with increased tumor cell death, suggesting Lyn is important for tumor survival *in vivo*. This is consistent with a prior report that dasatinib inhibits malignant glioma growth in a mouse model [8]. However, dasatinib used as a single agent in GBM patients who had failed therapy with Avastin® did not result in either a partial or complete response [48]. The reason for this is unclear but may be related to the diverse effects of dasatinib, *e.g.*, in addition to blocking the function of all SFKs it also targets the cytoplasmic non-receptor tyrosine kinase c-Abl, which is necessary for apoptosis of brain microvessel endothelial cells induced by an integrin αvβ3/αvβ5 inhibitor and by latrunculin [49]. Currently, it is not possible to conclude that nutrient deprivation is the trigger of the effects of Lyn *in vivo*, but a focal lack of nutrients does occur in GBM [2].

Elevation of Lyn is associated with resistance to therapy. In CML, it is associated with the development of resistance to imatinib mesylate, a Bcr-Abl tyrosine kinase inhibitor [13], [14]. It also is found in AML refractory to therapy [15], [16], and in non-small cell lung cancer cells resistant to cetuximab (an anti-EGFR antibody) where Lyn promotes EGFR nuclear translocation [5]. In addition, Lyn cooperates with a CD44-variant receptor in promoting chemoresistance in colon cancer cells [43]. In cancers refractory to therapy, autophagy can be a pro-survival response to the stress of chemotherapeutic agents [22], [40]; however, autophagy can lead to cell death under certain circumstances (reviewed in [50]). There is an evolving concept that cross-talk between autophagy and apoptosis signaling pathways occurs in cells, and not infrequently these two processes appear to be regulated in an inverse manner [51]. Hydroxychloroquine, an inhibitor of autophagy, has been entered into a phase II clinical trial involving patients with multiple types of cancer including GBM (reviewed in [22]), and blockade of autophagy has been shown to result in the sensitization of prostate cancer cells, multi-drug resistant v-Ha-ras transformed NIH-3T3 cells and other cells to a SFK inhibitor in nutrient-rich conditions [11], [52].

In summary, we demonstrate the novel findings that Lyn promotes autophagy and proliferation, as well as inhibits cell death, in nutrient-deprived GBM cells in culture and *in vivo* using a mouse model. Lyn promotes the malignant phenotype of GBM and multiple other cancers, including promotion of the epithelial-mesenchymal transition in breast cancer [18], as well as chemoresistance in certain cancers. Moreover, a Lyn-specific peptide that inhibits Lyn-dependent phosphorylation has been shown to decrease prostate cancer growth and induce apoptosis *in vivo* [53]. Thus, Lyn could be an important new therapeutic target for multiple cancers.

## Supporting Information

Figure S1 Inhibition of SFKs with PP2 inhibits the activity of CA-Lyn, and CA-Lyn promotion of autophagy in nutrient deprived SNB19 GBM cells. A, U87 GBM cells stably expressing CA-Lyn, DN-Lyn or the LV control were plated and then starved of L-glutamine and FBS as indicated in Figure 1A. A, PP2 (200 nM) or vehicle DMSO was added to the media after overnight starvation. On day 5 of starvation, cells were detergent lysed and immunoblotted with the indicated antibodies. B & C, SNB19 cells were plated in Hams F12 media with L-glutamine and 10% FBS, at 16 h washed and the media replaced with L-glutamine and FBS-free media with 1% BSA. B, After 48 hours of starvation, cells were lysed in NP40 lysis buffer with protease inhibitors; equivalent amount of lysate (100 µg) subjected to SDS-PAGE, and immunoblotted with the indicated antibodies, or C, after 7 days of starvation cells were detergent lysed and immunoblotted with the indicated antibodies. (TIF)

Figure S2 Inhibitors of autophagy block the survival of GBM cells in nutrient deprivation conditions. U87 GBM cells stably expressing CA-Lyn, DN-Lyn or the LV control were plated and then starved of L-glutamine and FBS as indicated in Figure 1A. After 20 hours of starvation, 3-MA or chloroquine were added and viable adherent cells counted by trypan blue exclusion on days 3 and 5. Representative fields were photographed on day 5 (A), and the data analyzed and presented as the mean±SEM (B). (TIF)

Figure S3 Effect of Rapamycin on LC3 protein and analysis of Akt and FAK activity in GBM cells expressing CA-Lyn or DN-Lyn. U87 GBM cells stably expressing CA-Lyn, DN-Lyn or the LV control were plated and then starved of L-glutamine and FBS as indicated in Figure 1A, or SNB19 GBM cells were plated in Hams F12 media with L-glutamine and 10% FBS, at 16 h washed, and the media replaced with L-glutamine and FBS-free media with 1% BSA. A, After 4½ days of starvation cells were treated with vehicle, 100 nM Rapamycin or 5 µM perfosine (overnight), followed by detergent lysis and immunoblotting with the indicated antibodies. All samples were electrophoresed on the same gel. B & C, On the indicated days of starvation, cells were detergent lysed and immunoblotted with the indicated antibodies. The normalized pAkt was determined based on the densitometric ratio of pAkt to normalized total Akt (Akt/GAPDH), and the normalized pFAK was determined based on the densitometric ratio of pFAK to normalized total FAK (FAK/GAPDH). (TIF)

Figure S4 Expression of CA-Lyn increased the autophagosome number/cell and the levels of pAMPK in nutrient-deprived glioma stem cells, and GFP expression in xenograft tumors indicates expression of the lentiviral construct. A-B, Human GSCs expressing CA-Lyn, DN-Lyn or LV were plated onto laminin-coated wells in NBM. After 24 h, the media was replaced with NBM lacking EGF and bFGF and 6 h later the cells were fixed, and reacted with anti-LC3 antibody followed by Alexa-594-conjugated secondary antibody and DAPI and visualized and photographed. Representative photomicrographs (scale bar 10 µm) are shown in (A). The number of red autophagosomes were counted in at least 25 cells with each construct. Data are presented as the mean ± SEM and analyzed using one-way ANOVA (B). It should be noted that the absolute number of autophagosomes per cell in the GSCs cannot be compared to those in the U87 GBM cells (Figure 3A & B) as the time of nutrient deprivation and the method used to detect the autophagosomes were different. C, Human GSCs expressing CA-Lyn, DN-Lyn or LV were plated and starved of EGF and bFGF as in panels A-B, whole cell lysates were then western blotted with the indicated antibodies. D, U87-LV, U87-CA-Lyn and U87-DN-Lyn expressing cells were harvested, resuspended in PBS, and 1×10^5^ cells in 5 µl injected with stereotactic assistance into the nude mouse brain. At 35 days, mice were euthanized, and the brains harvested, fixed and analyzed. Expression of the IRES-driven GFP gene in the lentiviral vector of LV, CA-Lyn and DN-Lyn is demonstrated by GFP immunohistochemistry. T, denotes tumor; and AMB, denotes adjacent mouse brain. (TIF)
