# Supplementary figures and images for: Lyn Facilitates Glioblastoma Cell Survival under Conditions of Nutrient Deprivation by Promoting Autophagy
Source: PLoS One. 2013 Aug 2;8(8):e70804. doi: 10.1371/journal.pone.0070804 (PMC3732228; doi:10.1371/journal.pone.0070804)

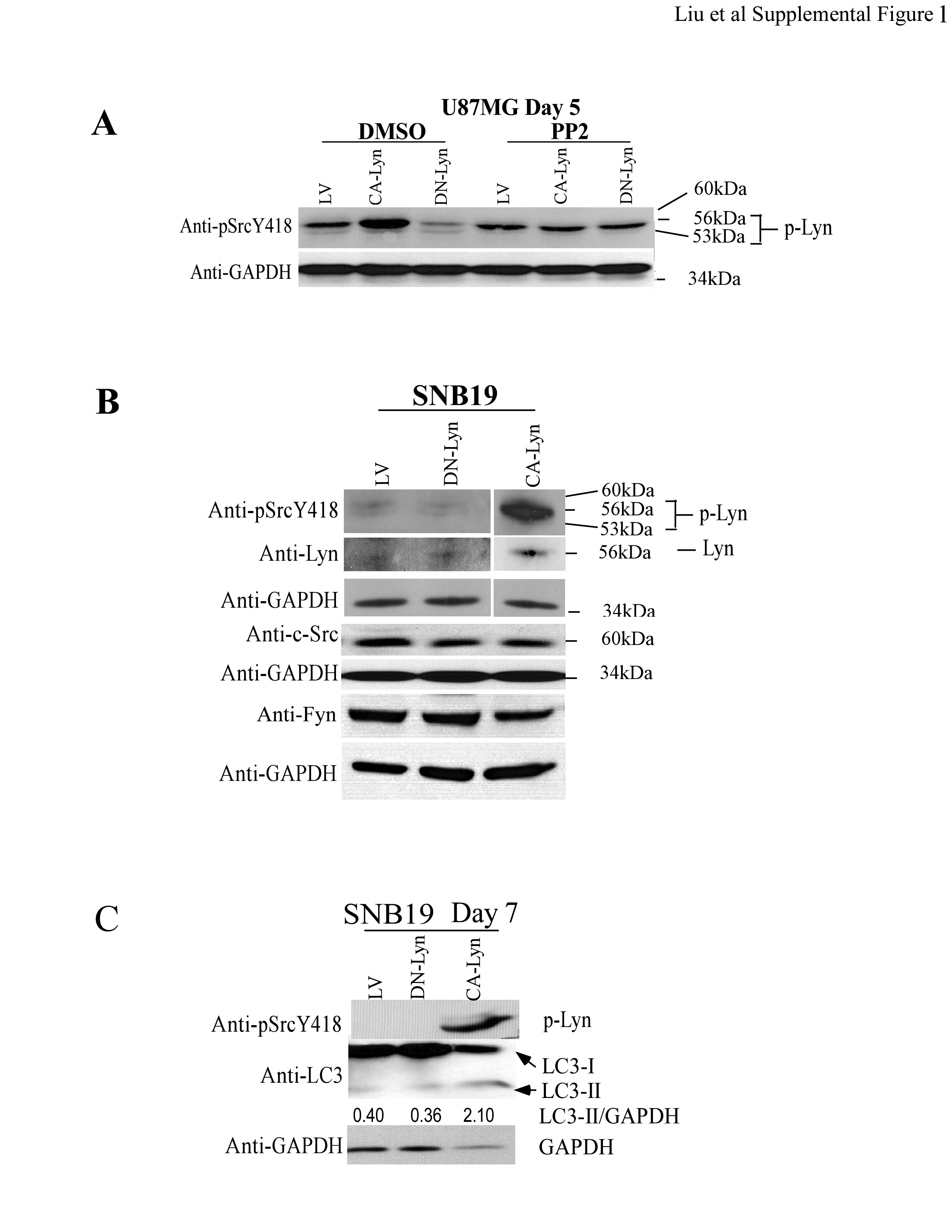

Supplement: Figure S1 — (TIF) [file pone.0070804.s001.tif]

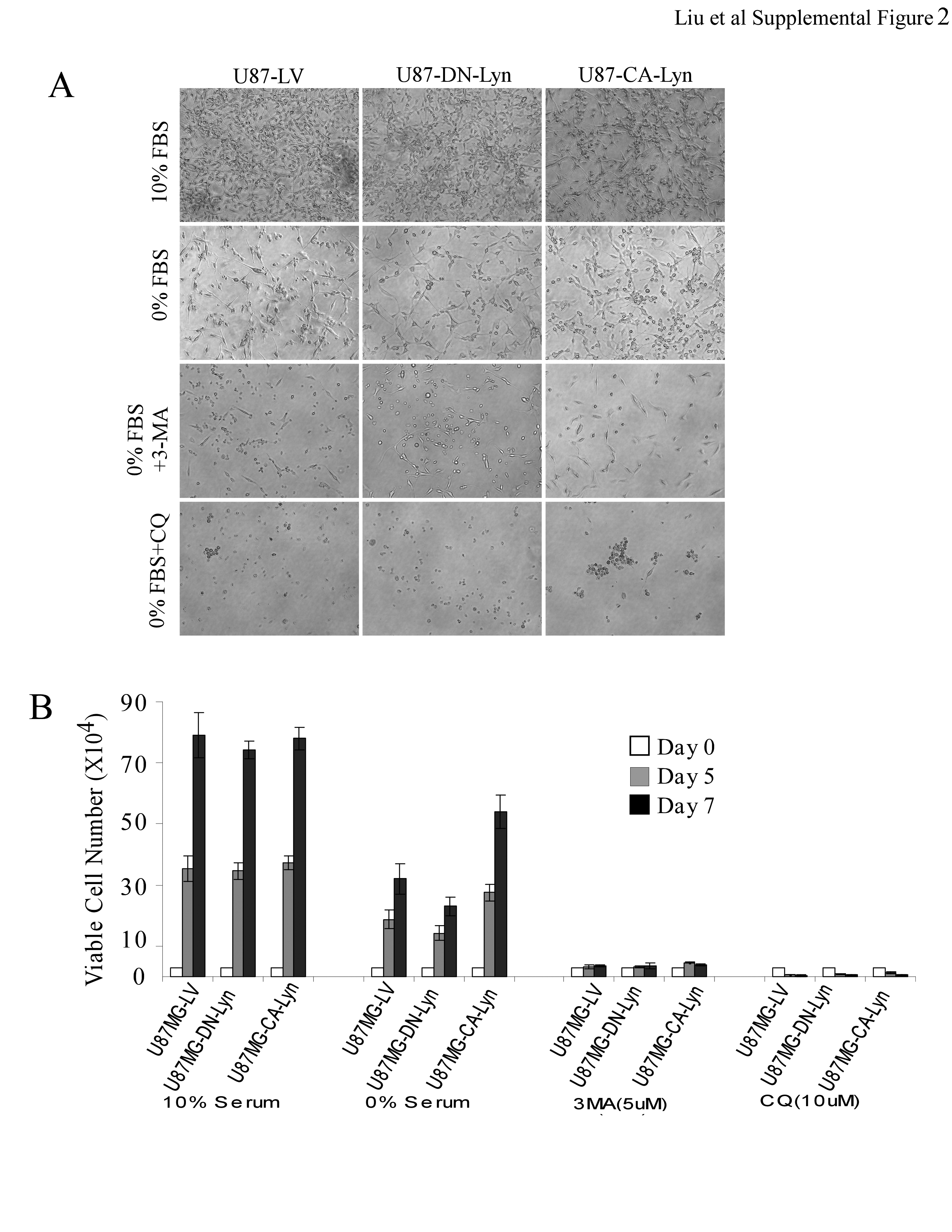

Supplement: Figure S2 — (TIF) [file pone.0070804.s002.tif]

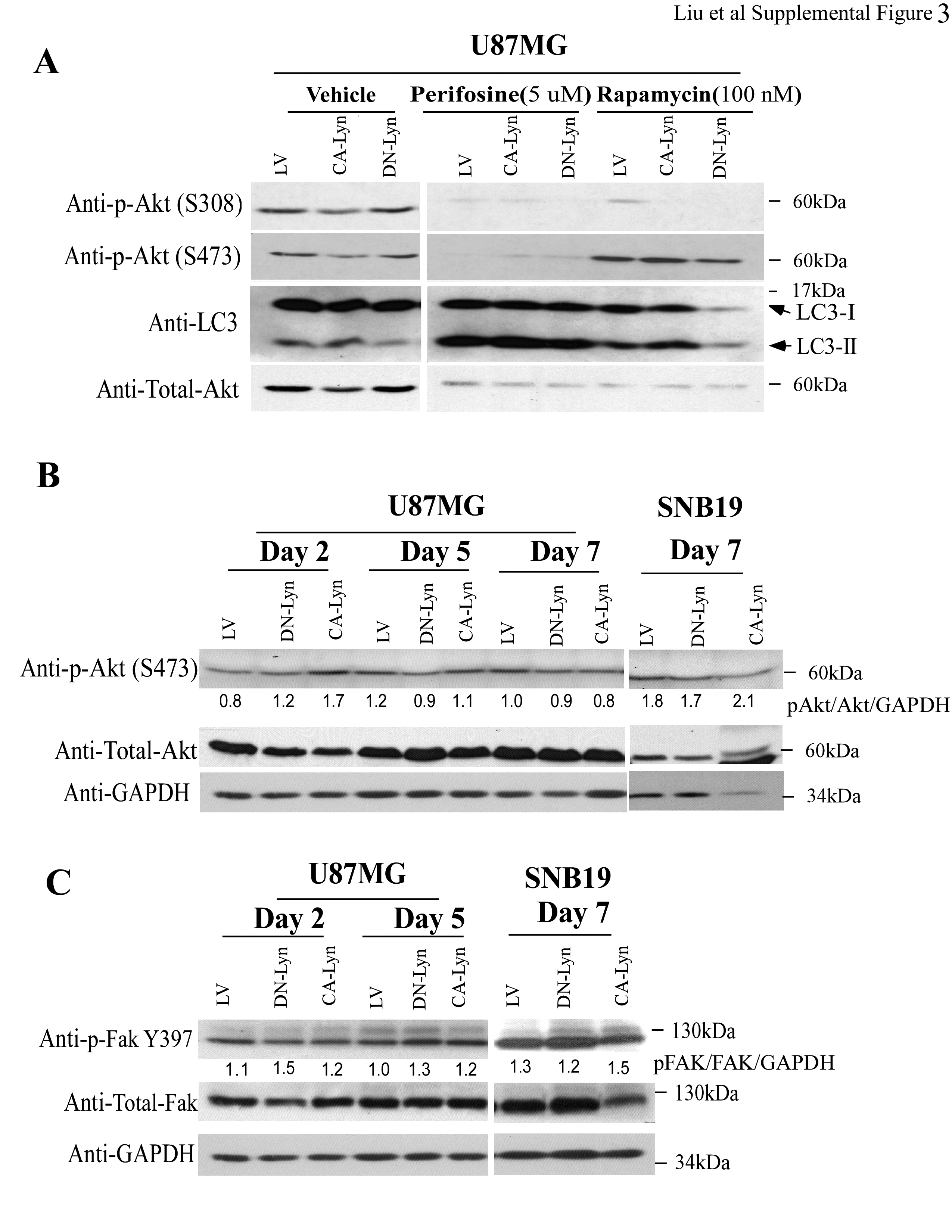

Supplement: Figure S3 — (TIF) [file pone.0070804.s003.tif]

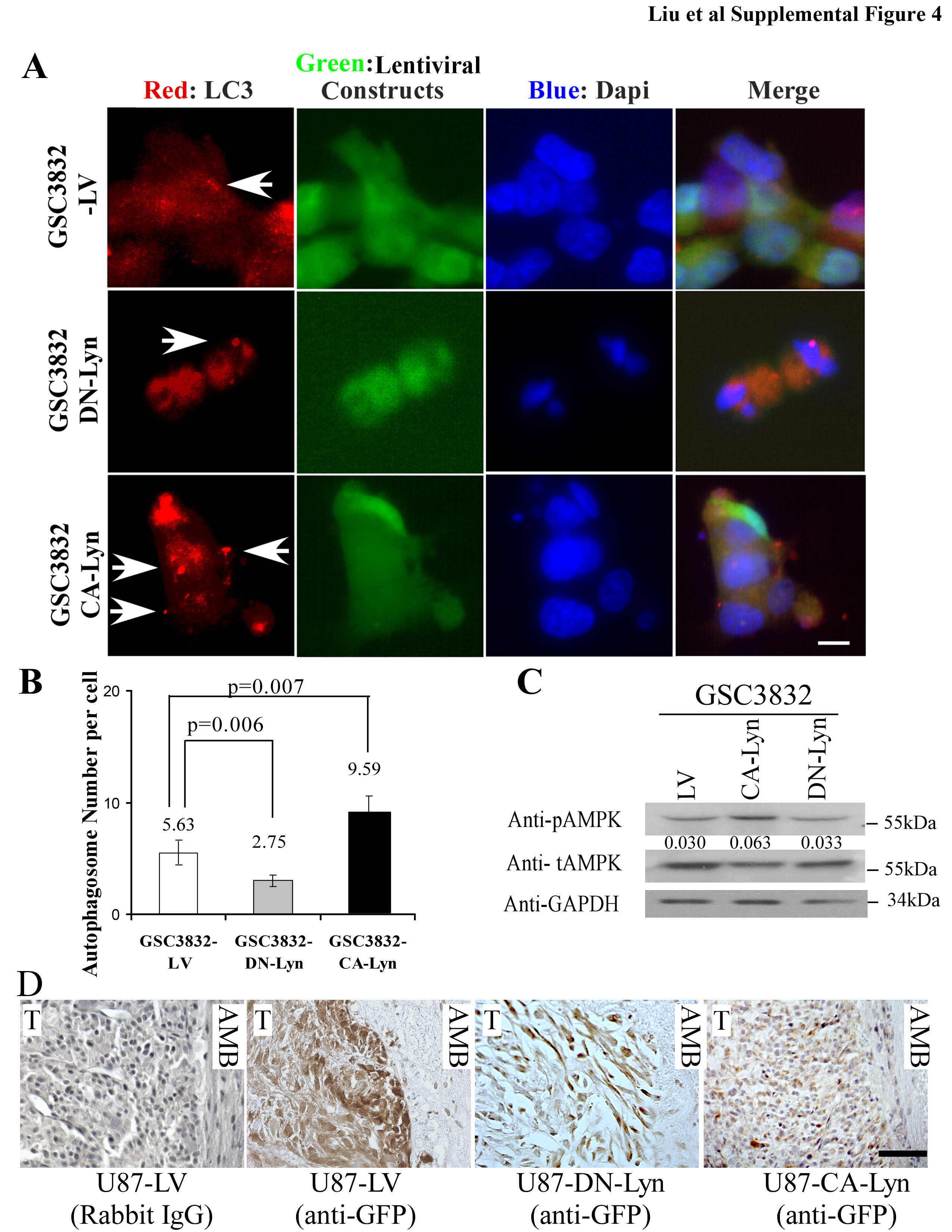

Supplement: Figure S4 — (TIF) [file pone.0070804.s004.tif]
